# Disseminated alveolar echinococcosis in a patient diagnosed by metagenomic next-generation sequencing: A case report

**DOI:** 10.3389/fpubh.2022.972619

**Published:** 2022-08-25

**Authors:** Junyan Qu, Huan Xu, Xiaoju Lv

**Affiliations:** ^1^Center of Infectious Disease, West China Hospital of Sichuan University, Chengdu, China; ^2^Pathology Department, West China Hospital, Sichuan University, Chengdu, China

**Keywords:** alveolar echinococcosis, next-generation sequencing, *Echinococcus multilocularis*, diagnose, albendazole (ABZ)

## Abstract

**Background:**

Alveolar echinococcosis (AE) is a parasitic zoonosis with high mortality and disability rates. Diverse clinical manifestations and mimicking of differential diagnoses such as tuberculosis and malignancy pose a diagnostic dilemma. With the rapid development of molecular diagnostic techniques in recent years, metagenomic next-generation sequencing (mNGS) has become an attractive approach for the etiological diagnosis of infectious diseases.

**Case presentation:**

we report a case of 51-year-old Chinese Tibetan male presented with 3-year low-back pain and 4-month discomfort in the right upper quadrant of the abdomen. He had been in good health. He was diagnosed with tuberculosis and was given anti-tuberculosis treatment a month prior to the visit, but the symptoms were not relieved. Abdominal computerized tomography (CT) revealed a hypodense lesion with uneven enhancement in the liver, and two ring-enhancing cystic lesions in the right abdominal wall. Lumbar spine enhanced MRI showed lesions of mixed density with uneven enhancement in the L1 vertebra and paraspinal tissue. The pathological results of the liver biopsy revealed parasitic infection and possibly echinococcosis. The metagenomic next-generation sequencing (mNGS) of the puncture fluid of abdominal cysts using Illumina X10 sequencer revealed 585 sequence reads matching *Echinococcus multilocularis*. Disseminated AE was diagnosed. Albendazole (400 mg, twice daily) was used, and the patient was in stable condition during follow-up.

**Conclusions:**

mNGS may be a useful tool for the diagnosis of AE. The case would help clinicians to improve their diagnostic skills.

## Background

Echinococcosis is a zoonosis, mainly including cystic echinococcosis (CE) and alveolar echinococcosis (AE), caused by *Echinococcus granulosus sensu lato* (sl) and *Echinococcus multilocularis*, respectively, with high mortality and disability rates (1). The incidence of AE is lower than that of CE, about 0.03 to 1.2 per 100,000 in endemic areas, but it has a high mortality and disability rate, and the mortality is more than 90% in untreated or inadequately treated patients within 10–15 years after diagnosis (2). The endemic areas of AE mainly include Switzerland, Alaska, Canada, eastern and central France, southern Germany, western China, and northern Japan (2–4). There are approximately 18,235 new cases of AE worldwide annually, of which about 91% cases occur in China (5). A recent epidemiological survey showed that the incidence of AE in western China was about 0.27% (4). The incidence of AE is high in the Tibetan Plateau and even higher than that of CE in several areas (6).

Clinical diagnosis of AE depends on epidemiological history, clinical presentation, radiographic findings and serology positive for AE. The diagnosis of AE is confirmed if histopathology compatible with AE or detection of *E. multilocularis* nucleic acid sequence(s) in a clinical specimen (7). However, there are false positives and false negatives in serological tests, and tissue specimens are sometimes unavailable. A study using molecular diagnosis showed that clinically diagnosed AE was misdiagnosed or unclassified in nearly 30% of cases (8). Molecular diagnosis was essential for the confirmation of AE. In recent years, due to the rapid development and substantially reduced costs of metagenomic next-generation sequencing (mNGS) technology, it has become an attractive approach for pathogen detection. mNGS also showed its advantages in the detection of parasites such as *Fasciola hepatica, Angiostrongylus cantonensis* and *Strongyloides stercoralis* (9–11).

Here we report a case of disseminated AE involving liver, subcutaneous tissue, and lumbar spine diagnosed by mNGS. This case highlights the challenges of diagnosing AE.

## Case presentation

**Figure 1:**
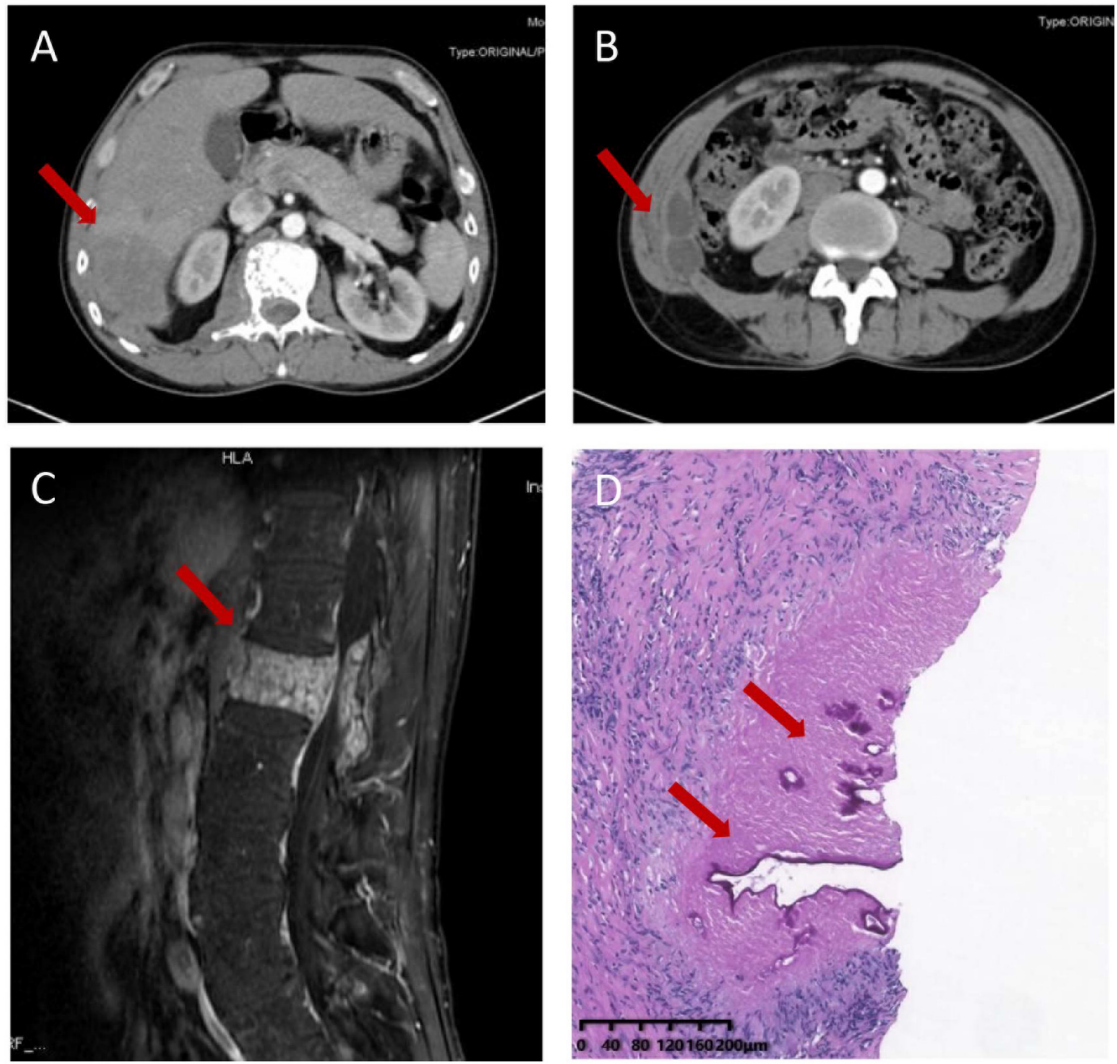
Imaging and pathological characteristics of the patient. Abdominal computerized tomography (CT) revealed a hypodense lesion with uneven enhancement in the right posterior lobe of the liver (6.5 × 4.6 cm) **(A)** and two ring-enhancing cystic lesions in the right abdominal wall **(B)**. Enhanced MRI of the thoracic and lumbar spine shows a mixed density lesion with uneven enhancement in the L1 vertebra and paraspinal tissue **(C)**. Liver biopsy sample showing granulomatous inflammation with necrosis and lamellar structure with positive periodic acid-Schiff positive staining **(D)**.

**Figure 2:**
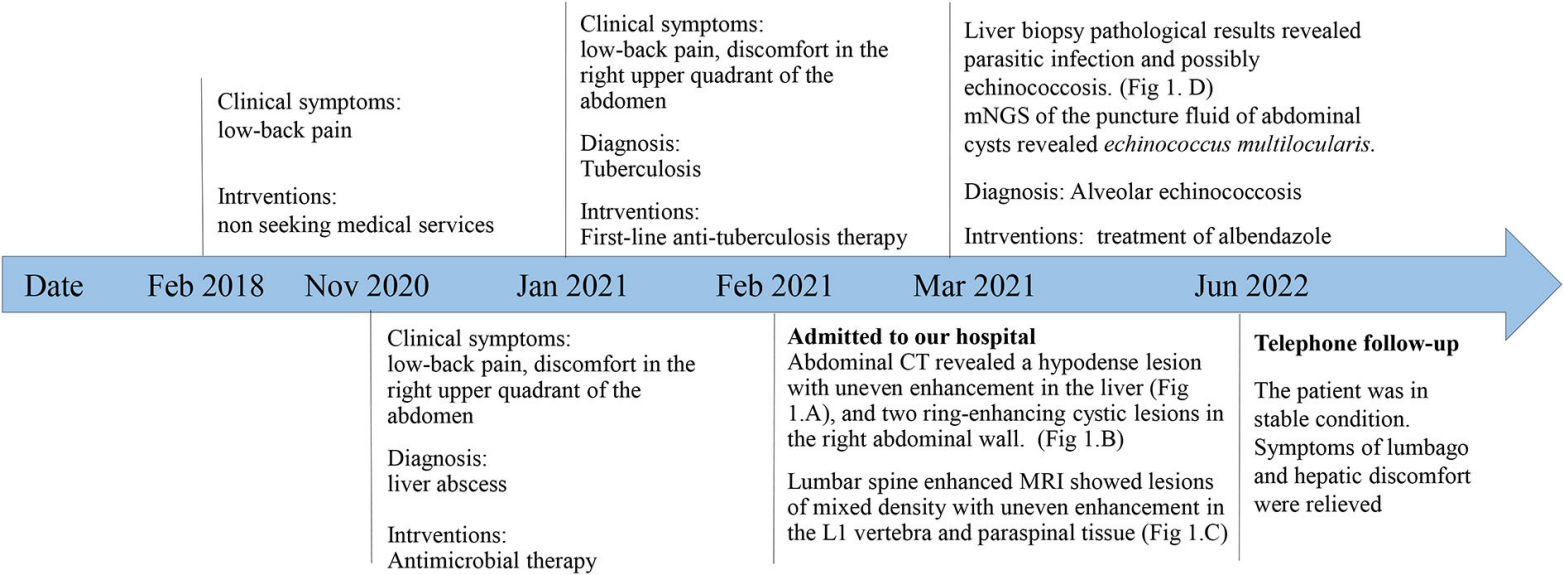
Timeline of the patient with relevant data of the past episodes and interventions.

A 51-year-old Chinese Tibetan male presented with 3-year low-back pain and 4-month discomfort in the right upper quadrant of the abdomen. He had always been in good health without alcohol abuse and underlying diseases. He was living in a rural area and had a history of occasional contact with dogs. Magnetic resonance imaging (MRI) of the lumbar spine in a local hospital suggested bone destruction in the L1 vertebra, slight swelling and thickening of the paravertebral soft tissue, and slight swelling of the right psoas muscle. He was clinically diagnosed with tuberculosis and was given anti-tuberculosis treatment a month prior to the visit, but the symptoms of low back pain were not relieved. On examination, his vital signs were normal. He has tenderness in the right upper quadrant. The percussion test of the lumbar spine was positive. Laboratory results showed an elevated C-reactive protein (CRP) concentration (52.4 mg/L) and erythrocyte sedimentation rate (83 mm/h). Both the interferon gamma (IFN-γ) release assay (IGRA) and PPD skin test were positive. The concentration of procalcitonin was 0.04 ng/ml. The complete blood count and concentrations of aspartate aminotransferase, alanine aminotransferase, total bilirubin, alkaline phosphatase, and lactate dehydrogenase were normal. IgG antibodies to echinococcus and cysticercus were positive. Tests for human immunodeficiency virus (HIV), (1,3)-β-D-glucan (BDG) and galactomannan were negative. Antibody tests for autoimmune hepatitis were negative. Abdominal computerized tomography (CT) revealed a hypodense lesion with uneven enhancement in the right posterior lobe of the liver (6.5 × 4.6 cm) that was suggestive of an abscess or tumor (Figure 1A). There were two ring-enhancing cystic lesions in the right abdominal wall (Figure 1B), which was connected to the intrahepatic lesion. Enhanced MRI of the lumbar spine showed lesions of mixed density with uneven enhancement in the L1 vertebra and paraspinal tissue, and the lesion spread upward along the right paraspinal to the right side of the T11 vertebra (Figure 1C). A brain MRI scan was normal. Chest CT revealed chronic bronchitis, emphysema, and bullae. The pathological results of the liver biopsy showed granulomatous inflammation with necrosis, periodic acid-Schiff (PAS) staining (Figure 1D) and silver hexamine immunohistochemical staining were positive, acid fast staining and tuberculosis real-time fluorescence quantitative PCR were negative, suggesting a diagnosis of parasitic disease and possibly echinococcosis. mNGS of the puncture fluid of abdominal cysts was performed using an Illumina X10 sequencer with a unilateral read length of 75 bp. After removing human sequences, there were 585 sequence reads matching *Echinococcus multilocularis*, but no reads matched any other parasites or microorganisms. He was diagnosed with disseminated alveolar echinococcosis. After consultation with a surgeon, it was determined that this case could not be treated surgically. Albendazole (400 mg bid) was administered orally. The patient was in stable condition during follow-up. The timeline of the patient with relevant data of the episodes is presented in Figure 2.

## Discussion

Western China, especially Qinghai-Tibet Plateau are the endemic regions of AE (4). Our patient came from Aba Tibetan and Qiang Autonomous Prefecture in Sichuan province, an echinococcosis epidemic area. A large epidemiological survey showed that more than 90% of AE patients here did not receive timely diagnosis and treatment due to poor economic conditions, inconvenient transportation and poor health service facilities and diagnostic technology (4). Diverse clinical manifestations and mimicking of differential diagnoses often result in misdiagnosis or missed diagnosis of AE.

The liver is the main organ involved in more than 95% of AEs. Extrahepatic involvement is rare and mainly spreads from the liver through lymphatic or hematogenous transmission to the lung, spleen, central nervous system (CNS), bones, lymph nodes or muscle (3). Bone involvement occurs in approximately 0.02–1% of AE cases, and vertebral involvement occurs in only a few dozen cases worldwide (3, 12). Although the initial symptom of our case was low back pain, there was a large lesion in the liver, which was connected to the lesions in the abdominal wall, and the vertebral lesions may be caused by hematogenous spread from the liver or direct infiltration from nearby lesions. The most commonly involved vertebrae were lumbosacral vertebrae in CE, while in AE, the most commonly involved vertebrae were the lower thoracic spine (T7-12), paravertebral space, and upper lumbar spine (L1-3) (3, 13), as in our patient.

The characteristic CT or T2-weighted MRI findings of liver AE are mainly multiple, indistinct and irregular low-density lesions (3, 14). Extrahepatic lesions have a multicystic, honeycomb appearance, with septations, and may be accompanied by calcification and/or rim enhancement (3, 15, 16). The spinal lesions presented vertebral infiltration and paravertebral abscesses. If calcifications are seen, echinococcosis is more frequently suggested. The differential diagnosis of vertebral AE includes vertebral tuberculosis, bacterial or fungal abscesses, and neoplasms. Spinal tuberculosis also occurs in the lower thoracic and lumbar vertebrae (17). The main manifestations of spinal CT or MRI are bone destruction, intervertebral disc involvement and paravertebral abscess (18). Intervertebral disc involvement is less common in spinal AE than in vertebral tuberculosis. This patient had multiple spinal bone destruction without intervertebral disc involvement, which may support the diagnosis of echinococcosis. Some new imaging techniques, such as perfusion-weighted MRI, diffusion-weighted imaging, 18F-FDG-PET-CT and 18F-FDG-PET-MRI, have also been applied in the diagnosis of AE in recent years (19–21).

AE patients with extrahepatic hematogenous metastases complicate the diagnosis. Laboratory tests are non-specific. Erythrocyte sedimentation rate (ESR) and C-reactive protein (CRP) may be elevated in patients with AE, and eosinophilia may occur in fewer patients. Our patient had normal eosinophil counts and elevated ESR and CRP. The serological methods conventionally used to distinguish AE from CE were ELISAs using purified and /or recombinant, or *in vitro*-produced *Echinococcus multilocularis* antigens (Em2/Em2+/Em18), which could identify 80–95% of cases (7). However, some studies have shown that the sensitivity of Em2-ELISA and Em18-ELISA in AE diagnosis were <50% (22). Moreover, these serological methods are not routinely available in most hospitals in China, as in our hospital. Hydatid antibody detection methods routinely obtained in Chinese hospitals cannot distinguish between *E. granulosus sl* and *E. multilocularis*. Because *Cysticercus cellulosae* and *E. multilocularis* have common antigens, serological tests for both hydatid and cysticercosis IgG may be positive, as in our patient, making differential diagnosis very difficult. An experienced pathologist can distinguish between AE and CE by histopathological examination. Histopathological findings of AE suggest Periodic-Acid-Schiff (PAS)-positive, narrower laminated layer and fibrosis, smaller cysts, with periparasitic granuloma and sparse lymphocyte inflammation (7). Pathological examination still leaves inconclusive diagnosis of AE (3). Histology-based diagnostic algorithms may be powerful in differentiating AE and CE (23).

Molecular diagnosis of AE by PCR or qPCR based on native biopsy material or formalin-fixed paraffin-embedded tissue samples is feasible and sensitive, especially in patients with atypical manifestations, extrahepatic localizations and immunosuppression (24, 25). As a rapidly developing molecular diagnostic technique, mNGS enables rapid detection and comprehensive identification of pathogens directly from clinical samples without prior presumption, with a higher sensitivity and accuracy. mNGS, in particular, has great advantages in the detection of emerging, hard-to-culture, atypical and rare pathogens, such as *Mycobacterium tuberculosis*, non-tuberculous mycobacteria, *Talaromyces marneffei*, and parasites (9, 10, 26). In one recent case report, the patient was diagnosed with AE by mNGS of CSF after multiple negative histopathological tests (27). Although our patient's histopathological findings were compatible with AE, the etiological diagnosis was ultimately dependent on mNGS. mNGS may be a useful tool to detect *E. multilocularis* in patients. mNGS may have a greater diagnostic advantage than histopathology for AE with extrahepatic involvement where tissue specimens are not readily available.

The treatment of AE should be decided by a multidisciplinary team consisting of a surgeon, radiologist, hepatologist, and infectious diseases physician. A combined surgical and medical approach is recommended for echinococcosis (7, 28, 29). The optimal treatment is a radical surgical resection with a safe margin of 2 cm. Percutaneous drainage associated with antibiotics are best adapted to the patients with central necrotic cavity complicated with bacterial/fungal infection. Perendoscopic procedures are best suited for patients with parasitic lesions that compress or block the bile ducts and are accompanied by jaundice and/or cholangitis. Palliative surgery should be avoided (30). Surgical resection is almost impossible in patients with disseminated AE, CNS involvement, or vertebral disease. Most vertebral AE patients underwent decompression to avoid paralysis, and a small number of patients underwent vertebral body resection. Puncture, aspiration, injection of hypertonic saline, and reaspiration cannot be recommended for AE (3). Albendazole is often recommended as a first choice for echinococcosis due to its high blood concentration (31). Mebendazole can also be used if albendazole is not well-tolerated. It has also been reported that in an AE patient with hematogenic subcutaneous and bone dissemination, disease progression occurred after 6 years of albendazole treatment, and the disease was stable after mebendazole treatment (32). All patients with inoperable and postoperative echinococcosis should be given long-term benzimidazoles treatment to limit the growth and metastasis of the lesion (7). *In vitro* studies of the tyrosine kinase inhibitor imatinib suggested that it may be a new treatment option for AE. However, its efficacy needs to be validated *in vivo* (33).

This study has some limitations. We did not obtain spinal histopathological or molecular biological diagnostic results, but this is more consistent with clinical practice. Our patient had evidence of AE from liver and subcutaneous masses. In addition, the lumbar spine imaging findings were similar to those of the published AE. Therefore, puncture was not performed for lumbar lesions in order to minimize trauma to the patient.

In conclusion, this case illustrates the challenges of diagnosing disseminated AE. With increasing use in some countries, such as China, mNGS may be a useful tool for the diagnosis of AE. This case may help clinicians to improve their diagnostic skills and reduce the misdiagnosis rate of AE.

## Data availability statement

The datasets presented in this study can be found in the following online repository: https://www.ncbi.nlm.nih.gov/sra/PRJNA860097; PRJNA860097.

## Ethics statement

The study involving human participants was reviewed and approved by the West China Hospital, Sichuan University. Written informed consent was obtained from the individual for the publication of any potentially identifiable images or data included in this article.

## Author contributions

XL and JQ designed the study. JQ and HX collected and interpreted the data. JQ drafted the manuscript. XL and HX modified the manuscript and finally approved the version to be published.

## Funding

This work was supported by 1•3•5 project for disciplines of excellence-clinical research incubation project, West China Hospital, Sichuan University (grant number: 2021HXFH032). The funders had no role in study design, data collection and analysis, decision to publish, or preparation of the manuscript.

## Conflict of interest

The authors declare that the research was conducted in the absence of any commercial or financial relationships that could be construed as a potential conflict of interest.

## Publisher's note

All claims expressed in this article are solely those of the authors and do not necessarily represent those of their affiliated organizations, or those of the publisher, the editors and the reviewers. Any product that may be evaluated in this article, or claim that may be made by its manufacturer, is not guaranteed or endorsed by the publisher.
